# Perillic Acid Synergizes With the Miltefosine Leishmanicidal Effect Against *Leishmania amazonensis*

**DOI:** 10.1111/pim.70102

**Published:** 2026-10-23

**Authors:** C. Ferreira, M. Paschoal, C. L. A. Passos, K. G. H. Silva, K. C. H. Silva, E. M. Saraiva, D. C. Soares

**Affiliations:** ^1^ Department of Immunology Paulo de Góes Microbiology Institute, Universidade Federal do Rio de Janeiro Rio de Janeiro Rio de Janeiro Brazil; ^2^ Laboratory of Functional Foods Institute of Nutrition Josué de Castro, Universidade Federal do Rio de Janeiro Rio de Janeiro Rio de Janeiro Brazil; ^3^ Hybrid Systems Laboratory, Faculty of Pharmacy Universidade Federal do Rio de Janeiro Rio de Janeiro Rio de Janeiro Brazil; ^4^ Laboratory of Surveillance and Biodiversity ‐ Universidade Iguaçu (UNIG) Nova Iguaçu Rio de Janeiro Brazil

**Keywords:** Leishmania, natural products, perillic acid

## Abstract

Perillic acid (PA), a monocyclic monoterpene derived from limonene, was evaluated for its leishmanicidal activity alone and in combination with miltefosine, as well as in its microemulsion‐based formulation. PA demonstrated synergistic activity with miltefosine and was effective against both promastigote and amastigote forms of *Leishmania amazonensis*. Its anti‐amastigote effect was independent of nitric oxide production but was partially associated with reduced arginase activity in stimulated macrophages. In a murine model of cutaneous leishmaniasis, topical treatment with a PA‐loaded microemulsion resulted in a marked reduction in parasite burden, achieving a 99% reduction in *L. amazonensis* infections. PA also showed efficacy in vivo across different administration routes and showed significant skin permeation when formulated in a microemulsion. These findings highlight PA as a promising leishmanicidal agent and support its potential application in the treatment of cutaneous leishmaniasis.

## 1 Introduction

Leishmaniasis is a neglected disease that affects 98 countries on five continents. Approximately 50,000 to 90,000 cases of visceral leishmaniasis and 0.7–1 million cases of cutaneous leishmaniasis occur every year [1]. Pentavalent antimonials have been the first‐line drugs to treat leishmaniasis for more than 80 years, but these drugs have high toxicity and adverse side effects. These negative characteristics have led to patient withdrawal and an increased incidence of resistant parasite strains [1, 2, 3]. Alternative compounds, such as pentamidine, amphotericin B and paramomycin, are secondary options for the treatment of resistant cases despite their cytotoxicity [4, 5]. Miltefosine, the first approved oral treatment for leishmaniasis, has been used since 2002 for visceral leishmaniasis in India, achieving a high cure rate. However, variable efficacy of miltefosine against cutaneous leishmaniasis has been observed across geographic regions and this drug is also teratogenic [6, 7].

Perillic acid (PA) (Figure 1) is a monocyclic monoterpene metabolite of limonene and perillyl alcohol identified in several mammalian species, including humans [8, 9]. PA has been reported to inhibit the protein prenylating enzymes farnesyl transferase and geranylgeranyl transferase [10, 11], to cause cell cycle arrest at the G0/G1 phase [12, 13] and inhibit the proliferation of lung and colon cancer cells [14, 15, 16]. Moreover, PA inhibits the release of Herpes simplex virus from infected Vero cells [17].

**FIGURE 1:**
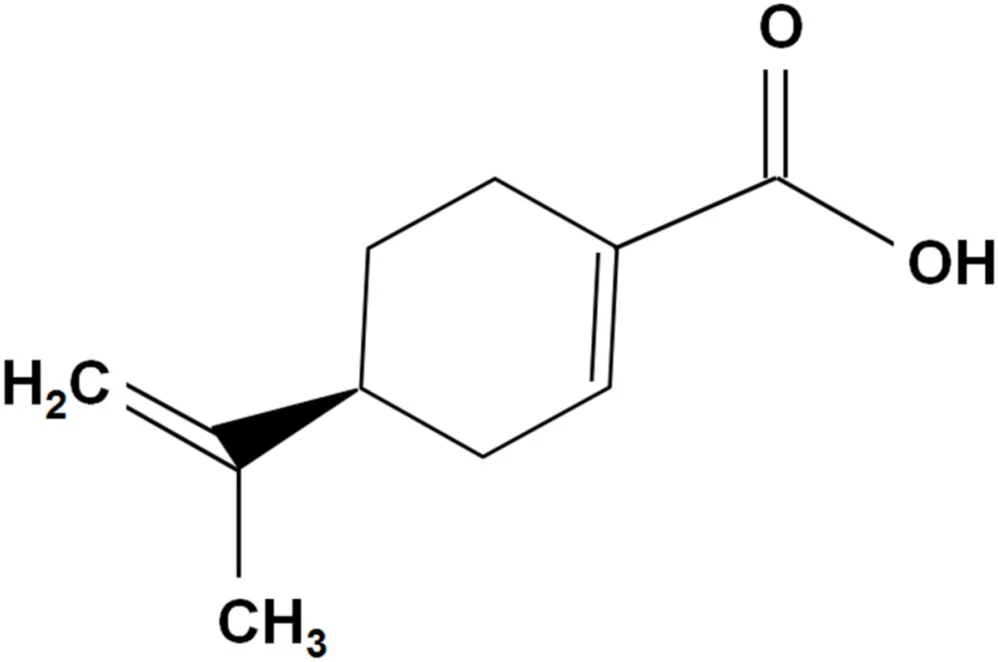
Chemical structure of perillic acid.

Microemulsions generally consist of water, oil, surfactant and in most cases, a cosolvent. Contrary to macroemulsions, microemulsions are transparent and thermodynamically stable single‐phase systems that demonstrate higher permeation rates than conventional emulsions or other vehicles [18, 19]. In addition, microemulsions can have droplet sizes ranging from 10 to 100 nm and in general, these dispersed systems do not coalesce. Increased solubility of active pharmaceutical ingredients and the capacity to induce drug partitioning into the stratum corneum render microemulsions an important vehicle for transdermal/dermal drug delivery [20]. Recently, reports on microemulsion formulations designed for topical or transdermal application have demonstrated that microemulsions improve the delivery of prostaglandin E1, diclofenac, indomethacin and lidocaine [21, 22]. The increased penetration‐permeation efficiency of the drugs carried in MEs can be attributed to the presence of hydrophilic and lipophilic domains.

Here, we aimed to analyse the activity of unformulated and formulated microemulsion‐based perillic acid and its association with miltefosine against *Leishmania amazonensis (L. amazonensis)*, which, in humans, may cause cutaneous leishmaniasis, severe anergic diffuse cutaneous leishmaniasis and the visceral form of the disease [21].

## 2 Materials and Methods

### 2.1 Ethics Statement

Female BALB/c mice (8–10 weeks), obtained from the Federal Fluminense University, Rio de Janeiro, RJ, Brazil animal facility, were housed at the Federal University of Rio de Janeiro (Rio de Janeiro, RJ, Brazil) in a temperature‐controlled room (23°C ± 1°C) with a 12/12 h light/dark cycle and water and food ad libitum. All experiments with animals were performed in strict accordance with the Brazilian animal protection law (Lei Arouca number 11.794/08) of the National Council for the Control of Animal Experimentation (CONCEA, Brazil). This study protocol was approved by the Committee for Animal Use of the Federal University of Rio de Janeiro (Permit Number: 128/15).

Female mice were selected to minimize aggression‐induced injuries and stress during cohabitation, ensuring that skin lesion assessments accurately reflected the experimental infection and treatment outcomes without interference from fighting wounds [22].

### 2.2 Drugs

Perillic acid and DMSO were purchased from Sigma‐Aldrich (St. Louis, MO, EUA). Miltefosine (Cayman Chemical, USA) was donated by Dr. Norton Heise (Universidade Federal do Rio de Janeiro, RJ, Brazil). Amphotericin B was purchased from Cristália Produtos Químicos e Farmacêuticos (Brazil).

### 2.3 Parasite Cultures

*Leishmania amazonensis* (WHOM/BR/75/Josefa) promastigotes were cultured at 26°C in Schneider insect medium (Sigma), 10% foetal calf serum (Gibco‐BRL, MD, US) and 40 μg/mL gentamycin (Schering‐Plough, Rio de Janeiro, Brazil).

### 2.4 Anti‐Promastigote Activity

Promastigotes were incubated at 26°C in Schneider insect medium with 10% FCS in the presence of different concentrations of PA and/or miltefosine. Parasite survival was estimated by counting the viable, motile forms in a Neubauer chamber 24, 48, 72 and 96 h after the addition of the drugs. In all tests, medium alone (CTRL) and 1% DMSO were used as controls. All cultures were performed in triplicate and the results were expressed as percent growth relative to the controls [23].

### 2.5 Anti‐Amastigote Activity

Peritoneal macrophages from female BALB/c mice obtained after stimulation with 3% thioglycolate for 3 days were harvested in RPMI 1640 medium (Biochrom KG, Germany). Macrophages were plated onto coverslips and allowed to adhere for 2 h at 37°C and 5% CO_2_. Nonadherent cells were removed by washing with 0.01 M phosphate‐buffered saline (PBS), and the macrophages were incubated overnight in RPMI with 10% FCS. The adhered macrophages were infected with *L. amazonensis* promastigotes (stationary phase) at a 10:1 parasite: macrophage ratio, incubated for 1 h at 34°C and 5% CO_2_ and then maintained for 24 h at 37°C and 5% CO_2_. The amastigote‐infected macrophage cultures were then treated with perillic acid and/or miltefosine in different concentrations for an additional 24 h. The cells were then washed, Giemsa‐stained and the number of amastigotes and the percentage of infected macrophages were determined by counting at least 200 cells in duplicate cultures. The infectivity index was obtained by multiplying the percent of infected macrophages by the mean number of amastigotes per infected macrophage. The results are expressed as the percentage of amastigote survival, comparing the infectivity index of infected and untreated macrophages [6].

### 2.6 Assessment of the Intracellular Load of *Leishmania amazonensis*

Infected macrophages were treated with 200 μM PA for 24 h or left untreated. They were then washed and incubated with 200 μL of 0.01% sodium dodecyl sulfate (SDS) for 10 min, followed by the addition of 800 μL of Schneider's medium with 10% FCS and cultured at 26°C for 2 days. The relative intracellular load of viable *L. amazonensis* amastigotes was measured by the parasite‐rescue transformation assay [24].

### 2.7 XTT Assay

Mouse peritoneal macrophages adhered to 96‐well plates were treated with perillic acid for 24 h and the cell viability was determined by adding 0.5 mg/mL 2,3‐bis[2‐methoxy‐4‐nitro‐5‐sulfophenyl]‐2H‐tetrazolium‐5‐carboxinilide inner salt (XTT, Sigma) and 10 μM phenazine methosulfate (PMS, Sigma). After 3 h of incubation, the absorbance of the reaction product was measured at 450 nm in a SpectraMax Paradigm (Molecular Devices). The results were expressed as the percentage of viable cells compared to the untreated control [25].

### 2.8 Trypan Blue Exclusion Assay

BALB/c peritoneal macrophages adhered to 24‐well plates and were treated with PA. After 24 h of treatment, 0.03% Trypan blue (Tetrasodium 3,3′‐[(3,3′‐dimethyl[1,1′‐biphenyl]‐4,4′‐diyl)bis(azo)]bis[5‐amino‐4‐hydroxynaphthalene‐2,7‐disulphonate]) was added and the number of viable cells was estimated by counting 200 cells in triplicate [24].

### 2.9 Phagocytosis

Female BALB/c peritoneal macrophages were treated with PA and incubated at 37°C in 5% CO_2_ for 24 h. Macrophages were then infected with *L. amazonensis* promastigotes at a 10:1 parasite: cell ratio for 1 h or 24 h and the rate of parasite‐macrophage association was determined by randomly counting at least 200 cells in each of duplicate coverslips [24].

### 2.10 Selectivity Index Calculation

The selectivity index was calculated as the ratio of murine peritoneal macrophages (CC_50_) to the IC_50_ obtained for *L. amazonensis* intracellular amastigotes.

### 2.11 Nitric Oxide Production

Thioglycolate‐stimulated peritoneal mouse macrophages obtained as described above (10^6^ cells/well in a 24‐well plate) were activated with 200 ng/mL recombinant mouse IFN‐γ (Bioscience Inc., CA, USA) or left untreated. After incubation for 24 h at 37°C in 5% CO_2_, the cells were treated with 200 μM PA. The nitrite concentration in the culture supernatants was determined by the Griess method. The absorbance was measured at 540 nm in a SpectraMax Paradigm (Molecular Devices) and the concentration of NO_2_^−^ was determined using a standard curve of sodium nitrite. The results were expressed as the micromolar concentration of nitrite [24].

### 2.12 Arginase Activity

Uninfected or infected macrophages were stimulated with 20 ng/mL IL‐4 or left unstimulated and treated with or without 200 μM PA, then lysed to determine arginase activity. The macrophage lysates were obtained from 10^6^ cells treated with 100 μL of 0.1% Triton X‐100 for 30 min, followed by the addition of 100 μL of buffer containing 25 mM Tris–HCl, pH 7.4 and 10 μL of 10 mM MnSO_4_. The enzyme was then activated by heating the lysate for 10 min at 56°C and arginine hydrolysis was performed by incubating 100 μL of the activated lysate with 100 μL of 0.1 M arginine (pH 9.7) at 37°C for 1 h. The reaction was stopped with 800 μL of H_2_SO_4_/H_3_PO_4_/H_2_O (1/3/7, v/v/v) and 40 μL of 10% isonitrosopropiophenone in 100% methanol, then heated to 100°C for 30 min. The urea concentration was measured at 540 nm. One unit of enzymatic activity was defined as the amount of enzyme that catalysed the formation of 1 μmol of urea per minute [23].

### 2.13 Fractional Inhibitory Concentration Index Determination

The fractional inhibitory concentration index (FIC) was calculated according to the following equation: FIC of PA = IC_50_ of PA in association/IC_50_ of PA alone. The same equation was applied to miltefosine. The sum of the FICs (ƩFICs) = FIC of PA + FIC of miltefosine, as described previously [26]. The mean of ƩFIC was calculated for each combination and then compared to the reference values and reported as synergism (xƩFIC ≤ 0.5), additivity (xƩFIC between > 0.5 and ≤ 4) or antagonism (xƩFIC > 4) [26, 27, 28].

### 2.14 Isobologram Analysis

The interaction between PA and miltefosine was analysed using FIC values to plot the isobologram according to the method of Tallarida [21]. The line connecting the two points is referred to as the line of additivity. Antagonistic combinations are defined as those points that lie above the line, and synergistic combinations are points that lie below the line [28].

### 2.15 Microemulsion

The microemulsion was composed of caprylic/capric triglyceride, polysorbate 80, glyceryl caprylate and distilled water in proportions sufficient to form an isotropic colloidal dispersion. The mixture was stirred to produce ultrasonic cavitation for 2 min at 40% amplitude. The droplet size was determined by dynamic light scattering, yielding 11.60 ± 0.023 nm. PA (2 mg/mL) was added to the microemulsion, followed by sonication for 15 min.

### 2.16 Evaluation of Perillic Acid In Vivo

Female BALB/c mice were infected subcutaneously with 10^6^ stationary‐phase *L. amazonensis* promastigotes in the left hind paw, and the assays were done once, as follows. Intraperitoneal treatment**—**1 week after infection, mice were randomly assigned to experimental groups: control without treatment (*n* = 4), DMSO (*n* = 4), PA (*n* = 4) and amphotericin B (*n* = 4). The treatment groups received 20% DMSO, 1 mg/kg/day amphotericin B or 50 mg/kg/day PA by the intraperitoneal route. All animals received a total of 10 applications on Days 7 to 11 and 14 to 18 after infection. On Day 28 after infection, the parasite load in the foot was determined by limiting dilution analysis as previously described [29]. Briefly, the suspension with the homogenized footpad was titrated using a 2‐fold serial dilution in 100 μL of Schneider medium supplemented with 10% foetal calf serum and incubated at 28°C for 7 days. The number of parasites was determined from the highest dilution at which promastigotes were detectable. Topical route**—**1 week after infection, mice were randomly assigned to experimental groups: control without treatment (*n* = 5), vehicle (drug‐free microemulsion formulation used as a control) (*n* = 8) and PA (*n* = 8). Treated groups received a microemulsion with or without PA at 1 mg/mL via the topical route. All animals received a total of 10 applications on days 10–14 and 17–21 after infection. On Day 25 post‐infection, the parasite load in the foot was quantified. Disease progression was evaluated weekly by recording the average paw diameter, calculated as the mean of the paw diameters in the horizontal and vertical directions. All assays were done in female mice to minimize aggression‐induced injuries and stress during cohabitation, ensuring that skin lesion assessments accurately reflected the experimental infection and treatment outcomes without interference from fighting wounds [22].

### 2.17 Measurement of the Blood Levels of AST and ALT in BALB/c Mice

Following treatment, blood samples were collected from all mice to measure aspartate aminotransferase (AST) and alanine aminotransferase (ALT) levels using quantitative kits (Doles, Goiânia, Brazil) according to the manufacturer's instructions.

### 2.18 Statistical Analysis

IC_50_ values were calculated following a nonlinear regression using a second‐order polynomial equation with a 95% confidence interval using GraphPad Prism 5 software. The data were analysed using Student's *t*‐test for two‐group comparisons or one‐way ANOVA for more than two groups in GraphPad Prism 5. *p* values equal to or less than 0.05 were considered significant.

## 3 Results

### 3.1 Anti‐Promastigote Activity

We first tested the activity of PA and miltefosine on the *p* *the proliferation of L. amazonensis promastigotes*. PA at concentrations of 100, 200 and 500 μM inhibited promastigote growth by 26.68%, 43.37% and 89.88% at 7 days post‐treatment, respectively, with an IC_50_ of 239.6 μM (Figure 2).

**FIGURE 2:**
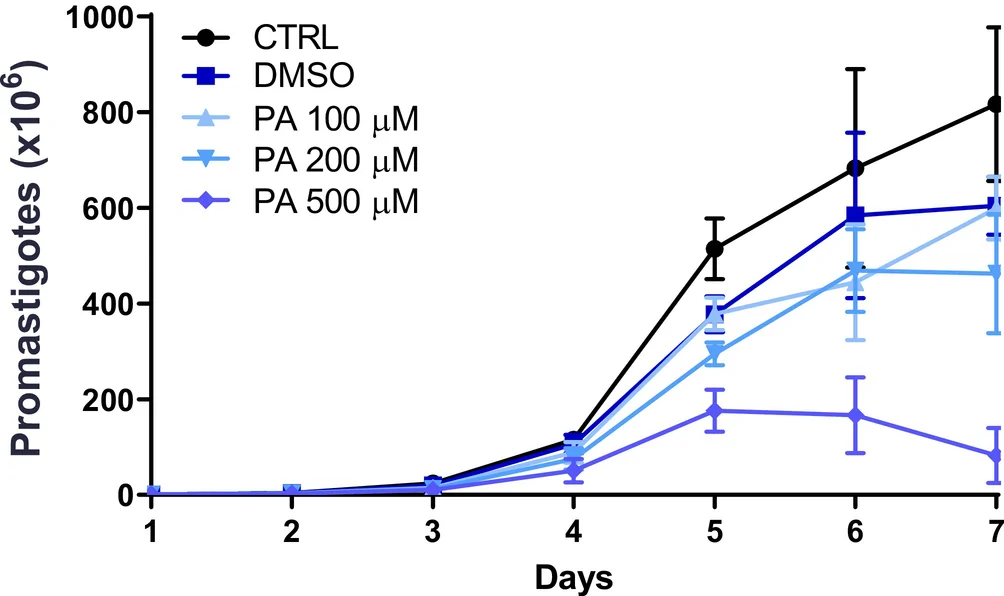
Effect of perillic acid on *L. amazonensis* promastigotes. Viable promastigotes were counted at the indicated times after treatment with different concentrations of perillic acid, vehicle (DMSO 1%) or nontreated (control). Data from 3 independent experiments are expressed as the mean ± SEM. **p < 0.01; ***p < 0.001 compared to the control.

### 3.2 Host Cell Viability Assays

To assess PA safety in host cells, we measured macrophage metabolic activity using the XTT assay. Our results showed that treating macrophages with PA up to 200 μM did not significantly affect their metabolic activity (Figure 3A). However, 1% sodium azide, a toxic compound, reduced macrophage viability by 83%.

**FIGURE 3:**
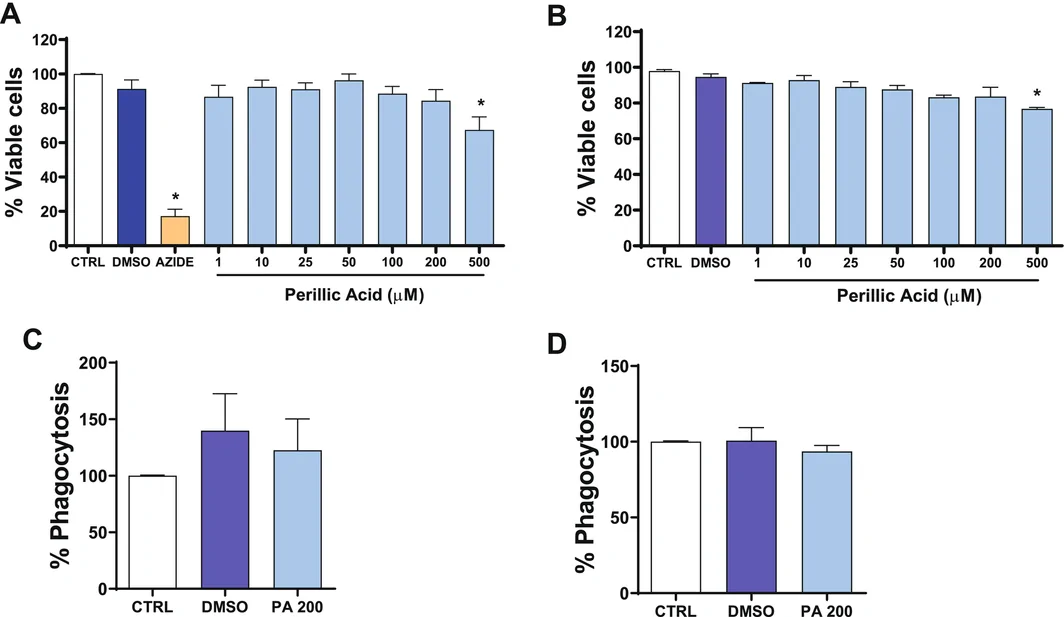
Cytotoxicity of perillic acid against murine peritoneal macrophages in vitro. (A) Cells were cultured with the indicated concentrations of perillic acid for 24 h at 37°C in 5% CO_2_. Sodium azide (1%) was used as a positive control. Viability was measured by the XTT method. (B) Macrophages were cultured for 24 h at 37°C in 5% CO_2_ with different concentrations of perillic acid and their viability was measured using the Trypan blue exclusion assay. (C, D) Peritoneal macrophages were treated with 200 μM perillic acid for 24 h and then incubated with promastigotes at a 1:10 ratio. After (C) 1 h and (D) 24 h, the cells were fixed and stained and the phagocytic rate was determined by counting at least 200 cells. The results are representative of 3 experiments performed in triplicate ± SEM. *p < 0.05 compared to the control.

Next, we tested PA's capacity to compromise macrophage membrane integrity using a Trypan blue dye exclusion assay. Our results showed that 77% of cells remained viable after treatment with 500 μM PA, compared with the untreated control (Figure 3B). DMSO, the diluent vehicle of PA, also did not damage the macrophage membranes.

Finally, we assessed phagocytosis, a functional property of macrophages, as a criterion of cell health. For this assay, *L. amazonensis* promastigotes were provided as particles for phagocytosis by murine macrophages that had been treated with or without PA. We observed that 200 μM PA did not affect phagocytosis of the promastigotes compared to untreated control cells at 1 h (Figure 3C) and 24 h post‐incubation (Figure 3D).

### 3.3 Antiamastigote Activity

We then evaluated PA activity against amastigotes, the stage that maintains the infection in vertebrate hosts. Thus, parasite killing was assessed in vitro in amastigote‐infected macrophages after 24 h of treatment. Our results showed that treatment with 1, 10, 25, 50, 100 and 200 μM PA killed 22%, 28%, 36%, 45%, 59% and 61% of the amastigotes, respectively. The IC_50_ of PA was calculated to be 63.8 μM (Figure 4A). Treatment of amastigote‐infected macrophages with 1, 5, 10, 20 and 40 μM miltefosine resulted in the killing of 9%, 26%, 48%, 56% and 74% of the parasites, respectively, with an IC_50_ of 14.08 μM (Figure 4B).

**FIGURE 4:**
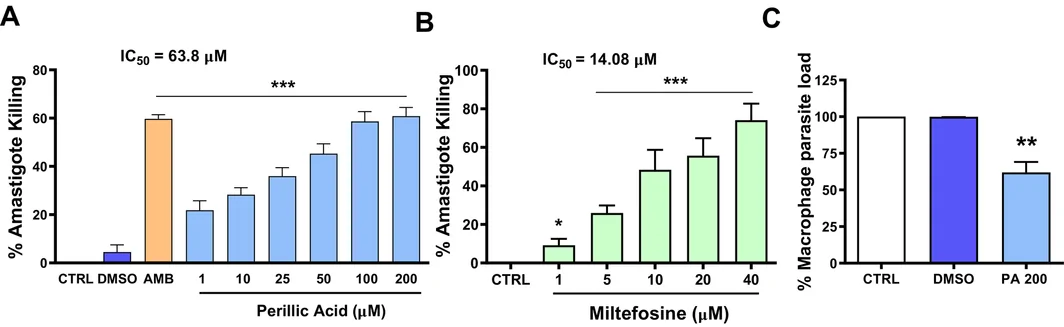
Leishmanicidal effects of perillic acid and miltefosine on amastigote‐infected macrophages in vitro. Peritoneal macrophages (10^5^) from BALB/c mice were infected with promastigotes at a ratio of 10 parasites to 1 macrophage for 24 h and were either left untreated or treated with the indicated concentrations of (A) perillic acid or miltefosine in 1% DMSO. The results are presented as mean ± SEM from 3 experiments performed in triplicate. (CB) Infected macrophages were left untreated or treated with 200 μM perillic acid for 24 h, washed, fed with Schneider's complete medium and cultured at 26°C. The macrophage parasite load was evaluated after 48 h in culture by counting promastigotes. The results are presented as mean ± SEM from 3 experiments performed in triplicate. *p < 0.05, **p < 0.01, ***p < 0.0001, compared to the control.

To further confirm the parasite‐killing effect, amastigote‐infected macrophages, treated with or without 200 μM PA, were cultured under conditions that promoted amastigote‐to‐promastigote transformation. The viable amastigotes that survived the treatment transformed into motile promastigotes, which were counted after 48 h of culture at 26°C (Figure 4C).

### 3.4 Fractional Inhibitory Concentration Index and Isobologram Analysis

Combination therapy is an important strategy for treating many diseases. Therefore, we assessed whether PA could synergize with miltefosine using the fractional inhibitory concentration index (FIC) and the isobologram method. The previously determined IC_50_ values for each compound tested alone were used as references to calculate the FIC values. The FIC for each drug was calculated by dividing the compound's IC_50_ in combination by its IC_50_ when tested alone. These values were plotted in an isobologram, with the FIC of miltefosine on the *x*‐axis and the FIC of PA on the *y*‐axis for amastigotes (Figure 5). The line connecting the points on the two axes represents the additivity line: points on it indicate additive effects, points above it indicate antagonism and points below it indicate synergistic interactions. The combination of PA with miltefosine produced experimental points below the additivity line, with a mean ΣFIC of 0.493 ± 0.228, confirming synergistic activity against amastigotes (Figure 5).

**FIGURE 5:**
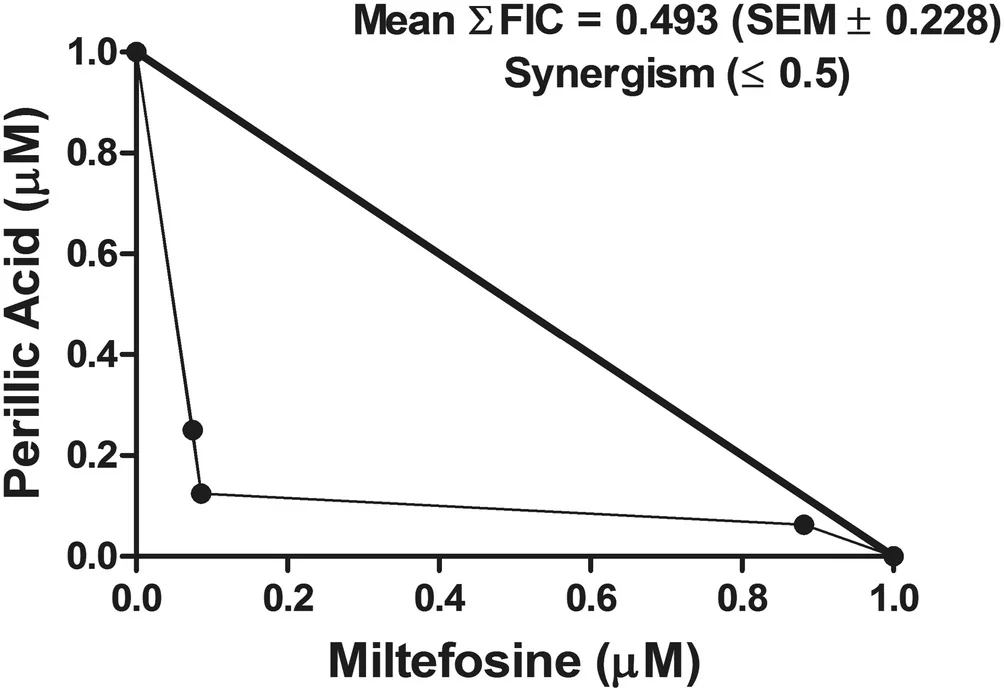
Leishmanicidal effects of the association of perillic acid and miltefosine. Isobolographic analysis of the killing effect for *L. amazonensis* amastigotes of the perillic acid‐miltefosine association. The straight line represents all additive theoretical combinations that should inhibit survival by 50%. The points below the additivity line represent synergistic combinations. The results are presented as mean ± SEM from three experiments performed in triplicate. FIC = fractional inhibitory concentration.

### 3.5 Nitric Oxide Production

NO is an important mediator of *Leishmania* death; therefore, we investigated whether PA could modulate macrophage NO production. Our results showed that PA did not induce NO production by infected or uninfected macrophages or by uninfected and infected macrophages stimulated with 200 ng/mL IFN‐γ, indicating that the antileishmanial effect of PA was not mediated by the induction of NO (Figure 6A).

**FIGURE 6:**
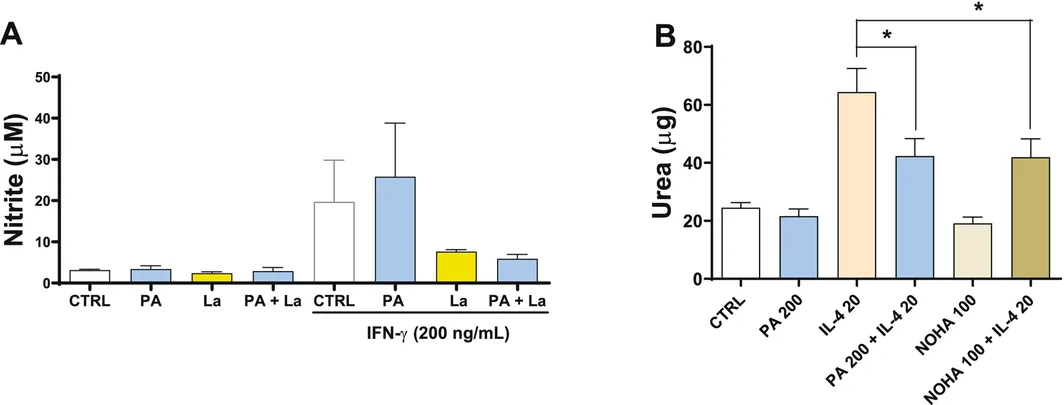
Effects of perillic acid on nitric oxide (NO) production and arginase activity by murine peritoneal macrophages. (A) Uninfected (10^5^) and *L. amazonensis*‐infected macrophages at a 10:1 ratio, stimulated with IFN‐γ or left unstimulated, were incubated in the presence or absence of 200 μM perillic acid. NO production was evaluated at 48 h post‐treatment using the Griess method. The results of three independent experiments performed in triplicate are shown as the mean ± SEM of nitrite concentration. (B) Macrophages (10^6^) were stimulated with 20 ng/mL IL‐4 or left unstimulated and then treated with 200 μM perillic acid or 100 μM NOHA, an arginase inhibitor, for 48 h. The results are presented as mean ± SEM from 3 experiments performed in triplicate. *p < 0.05, compared to IL‐4.

### 3.6 Evaluation of Macrophage Arginase Activity

To assess the effect of PA on the arginine pathway, we determined the arginase activity in murine peritoneal macrophage lysates that had been treated with PA or not for 48 h. Our results showed that, in the absence of a stimulus, 200 μM PA did not significantly reduce arginase activity relative to the untreated control. However, the arginase activity after stimulation with 20 ng/mL IL‐4, an arginase inducer, decreased by approximately 1.52‐fold after treatment with 200 μM PA (Figure 6B). N‐hydroxy‐L‐arginine (NOHA), a moderate inhibitor of arginase, showed a decrease in arginase inhibition of approximately 1.54‐fold (Figure 6B).

### 3.7 In Vivo Antileishmanial Activity

Next, the leishmanicidal effect of PA was assessed in vivo. Our results showed that treatment of infected mice with PA by the intraperitoneal route after 28 days post‐infection decreased the lesion size by 35.7% in comparison to the control, while PA treatment by the topical route reduced the lesion size by 38% in comparison to the control at the same time (Figure 7A,B,D,E). Importantly, PA inhibited 99% of the parasite load in the paw treated by both the intraperitoneal and topical routes in comparison to both control and vehicle‐treated mice (Figure 7C,F). Similar to PA, amphotericin B inhibited parasite growth by 99% after intraperitoneal treatment (Figure 7C). Amphotericin B was included as the reference drug in the in vivo assay because it is a well‐established standard of care for cutaneous leishmaniasis in murine models and also minimises variability in oral bioavailability and systemic toxicity commonly associated with miltefosine. Intraperitoneal treatment with perillic acid, DMSO and amphotericin B did not alter blood AST and ALT levels in mice (data not shown).

**FIGURE 7:**
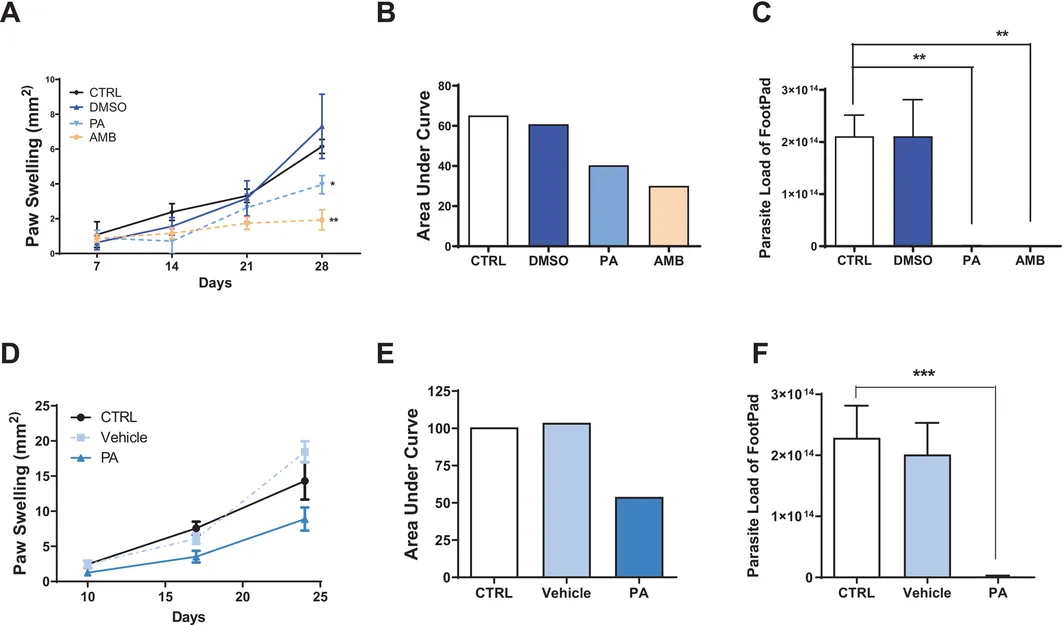
Effectiveness of perillic acid in the treatment of murine cutaneous leishmaniasis. BALB/c mice were infected with *L. amazonensis* in the left foot pad. (A) Treated groups of 4 animals receiving, by intraperitoneal route, 20% DMSO, 1 mg/kg/day amphotericin B (AMB) or 50 mg/kg/day perillic acid (PA). All animals received a total of 10 applications on days 7–11 and 14–18 after infection. Disease progression was evaluated weekly by recording the average paw diameter, calculated as the mean of the paw diameters in the horizontal and vertical directions. (B) Area under the curve of graph A. (C) The parasite load in the foot was verified 28 days post‐infection. (D) Treated animals receiving, by topical route, microemulsion with or without 1 mg/mL perillic acid. All animals received a total of 10 applications on days 10–14 and 17–21 after infection. Disease progression was evaluated as above. (E) Area under the curve of graph D. (F) On day 25 after infection, the parasite load in the foot was verified. *p < 0.05, **p < 0.01, ***p < 0.0001, compared to the control. Intraperitoneal treatment groups: Untreated control (*n* = 4), vehicle (*n* = 4), perillic acid (*n* = 4) and AMB (*n* = 4). Topical treatment groups: Untreated control (*n* = 5), vehicle (*n* = 7) and perillic acid (*n* = 8).

## 4 Discussion

In our study, we demonstrated that PA, a major metabolite of D‐limonene, a naturally occurring monoterpene [30], was active against *L. amazonensis* infection both in vitro and in vivo. To elucidate the effects on parasite growth, we first evaluated the effect of PA on promastigotes, showing that from the fifth day of incubation, PA inhibited promastigote growth by 90%. Importantly, PA was more effective against intracellular amastigotes, with an IC_50_ of 63.8 μM after a single 24 h treatment.

Miltefosine, originally developed as an anticancer drug, is the first oral treatment of visceral leishmaniasis [31]. This drug achieved remarkable cure rates (greater than 90%) for visceral leishmaniasis and has been shown to be effective in New World cutaneous leishmaniasis [32, 33]. However, a spread of Brazilian *Leishmania infantum* carries a homozygous deletion associated with reduced susceptibility to Miltefosine [34, 35]. Teratogenicity and a rather narrow therapeutic window in clinical trials are also limitations of this drug [12, 31]. Our data show that miltefosine presented an IC_50_ of 14.08 μM against intracellular *L. amazonensis* amastigotes after 24 h of treatment. Combination therapy is an interesting approach to decrease toxicity, reduce the duration and cost of treatment and increase the lifetime of old and new drugs [36, 37]. Several combined treatments for visceral leishmaniasis have been tested with positive results, leading to reduced adverse symptoms and a shorter duration of therapy [38]. It has been reported that the multimodal therapy associating miltefosine plus topical photodynamic therapy mediated by liposomes containing chloroaluminum phthalocyanine showed greater efficacy in reducing the paw diameter, number of parasites per paw and parasite viability in C57BL/6 mice infected with *L. amazonensis* [38]. In clinical practice, miltefosine is used in combination with liposomal amphotericin B or paromomycin to treat visceral leishmaniasis caused by *L. donovani* and overcome miltefosine's limitations; these combinations achieve efficacy rates of 96% and 98%, respectively. Our data also showed a potentiating effect on miltefosine's leishmanicidal activity when combined with perillic acid. A synergistic effect against *L. amazonensis* amastigotes, with a ΣFIC of 0.493, suggests that PA is an important compound for further studies of miltefosine combination therapy.

To evaluate the cytotoxicity of PA for the host cells, we performed XTT and Trypan blue assays. We did not observe cytotoxicity in peritoneal macrophages at concentrations up to 500 μM PA. Additionally, PA does not affect macrophage phagocytic capacity. Corroborating this result, it has been demonstrated that D‐limonene, a PA precursor, increased macrophage phagocytosis and microbicidal activity in lymphoma‐bearing mice without affecting macrophages from normal mice [38]. In addition, PA treatment did not alter blood AST and ALT levels in the mice.

The leishmanicidal mechanisms activated by PA were tested by its ability to modulate NO production in murine macrophages. Our results demonstrated that PA does not induce NO production by macrophages (data not shown). Similarly, D‐limonene did not increase the nitrite concentration in BALB/c peritoneal macrophages, although PA and perillyl alcohol presented trends towards mild increases in NO production (not statistically significant) by peritoneal macrophages from tumour‐bearing mice [38]. Here, we show that arginase activity in IL‐4‐stimulated macrophages was inhibited by PA treatment, which may partially explain its leishmanicidal activity through its action on polyamine synthesis.

Microemulsions have proven effective for developing formulations containing natural substances for wound treatment. The topical application of microemulsions containing propolis extract and cinnamon oil has been shown to accelerate wound healing in the cornea and skin, respectively [39, 40, 41]. Mice infected with *L. donovani*, when treated intravenously with microemulsions containing amphotericin B, retain the efficacy of the commercial formulation [42]. Taken together, these data demonstrate that the development of microemulsions is a strategy for the topical drug treatment of cutaneous leishmaniasis. Here, we showed that PA‐loaded microemulsion reduced disease progression, as measured by the average paw lesion diameter, more effectively than intraperitoneal PA treatment. Both transdermal and intraperitoneal PA treatments reduced the parasite burden in the lesion to a similar extent. Importantly, the dose required for transdermal treatment was 50 times lower than that for intraperitoneal administration. Previous studies have demonstrated that female mice develop larger leishmaniasis skin lesions, harbour significantly more parasites exhibit increased parasite dissemination due to a shift towards Th2 immunity [43]. Therefore, the leishmanicidal efficacy of transdermal PA‐loaded microemulsion treatment in this severe leishmaniasis model is particularly noteworthy.

Topical treatment of cutaneous leishmaniasis is an important alternative to reduce therapy withdrawal and consequently reduce parasite resistance. The only topical treatment recommended for cutaneous leishmaniasis by the World Health Organization (WHO) is paromomycin plus methylbenzethonium ointment for 20 days, which shows cure rates for cutaneous leishmaniasis caused by *Leishmania major* of 67%–77% [1, 44]. Studies with paromomycin plus 0.5% gentamicin showed cure rates of 80% and 82% for leishmaniasis caused by *L. major* and *Leishmania braziliensis* , respectively [1]. Studies have shown that 2% amphotericin B in petrolatum resolved two cases of cutaneous leishmaniasis caused by *L. infantum* without relapse [45, 46].

## 5 Conclusion

Our results demonstrate that PA exhibits anti‐*L. amazonensis* activity both in vitro and in vivo. In addition, treatment with PA‐microemulsion via the transdermal route was more effective at reducing lesion size than intraperitoneal administration, although parasite load reduction was comparable between routes. Further research is required to fully explore the potential of microemulsions as delivery systems for topical antileishmanial therapies, particularly for PA. Overall, PA appears to be a promising compound for drug development and the design of new antileishmanial agents.

## Author Contributions

D.C.S. and E.M.S. conceived the study, designed the experiments and analysed data. D.C.S, E.M.S., C.F., C.L.A.P. and K.G.H.S. wrote the manuscript. M.P, C.F., C.L.A.P., K.C.H.S. and D.C.S. performed the experiments. All authors read and approved the final version of the manuscript.

## Funding

This work was supported by the Coordenação de Aperfeiçoamento de Pessoal de Nível Superior (CAPES) finance code 001, Fundação Carlos Chagas Filho de Amparo à Pesquisa do Estado do Rio de Janeiro (FAPERJ), grants # E‐26/201.136/2021 to EMS and E‐26/210.136/210.672/2024 to DCS, 204‐019/2024 and Conselho Nacional de Desenvolvimento Científico e Tecnológico (CNPq), grants # 310670/2021‐7.

## Conflicts of Interest

The authors declare no conflicts of interest.

## Data Availability

The data that support the findings of this study are available on request from the corresponding author. The data are not publicly available due to privacy or ethical restrictions.
